# Working beyond disciplines in teacher teams: teachers’ revelations on enablers and inhibitors

**DOI:** 10.1007/s40037-020-00644-7

**Published:** 2020-12-22

**Authors:** Stephanie N. E. Meeuwissen, Wim H. Gijselaers, Ineke H. A. P. Wolfhagen, Mirjam G. A. oude Egbrink

**Affiliations:** 1 School of Health Professions Education, Faculty of Health, Medicine and Life Sciences, Maastricht University, Maastricht, The Netherlands; 2 Department of Educational Development and Research, Faculty of Health, Medicine and Life Sciences, Maastricht University, Maastricht, The Netherlands; 3 Department of Educational Research and Development, School of Business and Economics, Maastricht University, Maastricht, The Netherlands; 4 Institute for Education, Faculty of Health, Medicine and Life Sciences, Maastricht University, Maastricht, The Netherlands; 5 Department of Physiology, Faculty of Health, Medicine and Life Sciences, Maastricht University, Maastricht, The Netherlands

**Keywords:** Health professions education, Integrated curricula, Interdisciplinary teacher teams, Team learning

## Abstract

**Introduction:**

Health professions education faces transitions from monodisciplinary to integrated education and from soloist teachers to interdisciplinary teacher teams. Interdisciplinary teamwork has been found complex and prone to conflict. Teachers’ perceptions of why some teams work and learn as a real interdisciplinary team and others do not are lacking in this setting. We studied the factors that teachers perceive as enabling and/or inhibiting interdisciplinary team learning.

**Methods:**

In this exploratory, qualitative study, we conducted 17 semi-structured, vignette-guided interviews with teachers recruited from diverse disciplines in undergraduate health professions programmes at Maastricht University, the Netherlands, through maximum variation sampling. Team learning research informed data collection and template analysis.

**Results:**

We identified three themes representing the factors that teachers perceived to influence interdisciplinary team learning: ‘alignment/misalignment with the educational philosophy’ (regarding personal attributes, tendencies and motivation), ‘leadership practices’ (encompassing team vision, responsibility and reflection), and ‘involvement in organisational processes’ (covering organisational decision-making, support and learning opportunities). For interdisciplinary team learning in development of integrated education, teachers emphasised their personal ability to move beyond disciplinary boundaries. Shared team leadership enabled the creation of a shared vision, shared responsibility, and team reflection. Lastly, teacher involvement in educational management, peer support and learning was considered important.

**Discussion:**

To work beyond disciplines in health professions education, teachers should take an interest in integrated education, share responsibility and work in an environment where people continuously learn from others. Organisations can facilitate this by involving teachers in decision-making processes and providing faculty development aimed to foster shared leadership and team reflection.

**Supplementary Information:**

The online version of this article (10.1007/s40037-020-00644-7) contains supplementary material, which is available to authorized users.

## Introduction

*Suppose a team consisting of a cardiologist, a pulmonologist, a psychologist, a physiologist, and an anatomist are developing a first-year medical course entitled ‘Shortness of Breath’. As it stands, the course starts with one week of anatomy, followed by three weeks of common cardiovascular cases, three weeks of basic pulmonology and two weeks of symptom perception and anxiety disorders, respectively. The programme director is unhappy: she wants the team to integrate the above topics to reflect real clinical practice. However, this would require team members to go beyond their disciplinary boundaries and explore others’ expertise, which makes them feel uncomfortable.*

Interdisciplinarity as a core feature of effective clinical work practices can no longer be ignored in modern healthcare settings. Although it has become an accepted standard in clinical work, ‘infusing’ interdisciplinarity into health professions education (HPE) requires substantial effort despite the importance of integrated teaching expressed in earlier reports [1–3]. Integrated teaching aims to interrelate discipline-based subjects for the development of learning activities reflecting modern practice [4] and to stimulate students’ holistic approaches to patient problems [5]. In the example above, the programme director called upon her team members to discuss and negotiate each other’s perspectives and build an integrated course together, putting them in an uncomfortable position: to develop an interdisciplinary perspective they needed to do more than merely contribute their own expertise. In such endeavour, disciplines can ‘win or lose’ importance, depending on whether individual contributions are negotiated or synthesised into something new.

Strategies to design integrated curricula imply an important role for interdisciplinary teacher teams [6, 7]. However, earlier HPE research has suggested that collaboration within interdisciplinary teacher teams is prone to relational conflict [8] when individual course contributions are compromised. Such conflicts can be avoided by emphasising the synergistic strength of the team’s collective effort, rather than singling out individual goals and contributions [9]. In various other areas and levels of education, team teaching has been used for decades. However, this has been presented as a pedagogic choice and used to describe an activity of like-minded individuals [10, 11]. In HPE practice, the problem is that interdisciplinary teams are treated largely the same as monodisciplinary teams. Although it is generally recognised that the way people cooperate influences design and implementation process outcomes [12], research into what makes interdisciplinary teacher teams in HPE successful is scant.

Management sciences research has shown that team success across a wide range of settings—from business to healthcare to education—depends on team members’ ability to learn from each other [13, 14]. The way team members use and build upon each other’s knowledge and experience, also coined ‘team learning behaviour’ [14, 15], is shaped by the quality of interactions and the working climate, which should preferably foster engagement in negotiations where different views are acknowledged [13]. However, for team members to actually co-construct knowledge and achieve team learning, they must first have the opportunity to share knowledge and experiences [16]. Team interactions ending in discussion, negotiation and integration of knowledge and views have been coined ‘constructive conflicts’. These constructive conflicts eventually lead to a new idea or shared understanding [17, 18]; suppose a microbiologist, pharmacologist and public health scientist together agree upon a relevant student case about antibiotics in which indications, mechanisms and threats are intertwined. A systematic review on team learning in organisations has provided important insights into the generic influencing factors which were all related to team dynamics, such as social cohesion and reflexivity (teams acting upon reflection) [19, 20]. These findings, however, essentially reflected business teams aiming at products, profits and performance, rather than academic teams pursuing educational quality.

Earlier team learning research in HPE has suggested that teams with members from different disciplines create their own patterns and habits [21]. While some operate in a fragmented or framework-guided way and merely share knowledge, performing as a scattered group of individuals [22], others function in a truly integrated manner, are committed to the whole course and reach constructive conflicts, resulting in better education [21]. It is not clear, however, why the latter teams show higher team learning levels. In this study, we therefore focused on the factors that potentially enable and inhibit team learning. More specifically, using the ‘team learning’ concept, we sought to understand how interdisciplinary teams achieve the necessary interaction that makes them successful in integrated HPE. To this end, we addressed the following research question: what factors do teachers perceive as enabling and inhibiting team learning by teams with teachers from different disciplines? For this purpose, we conducted vignette-guided, semi-structured interviews with members from various teacher teams.

## Methods

In this explorative study, which forms part of a broader qualitative research project on interdisciplinary teacher teams in undergraduate HPE [21], we conducted qualitative, semi-structured interviews with teachers. We used prompting vignettes to explore teachers’ perceptions on team learning, operating from a social constructivist stance [23]. This position acknowledges findings’ socially and experientially based nature. The interactions in our interviews served to accumulate information and increase sophistication for a better understanding of these factors. We iteratively collected, analysed and interpreted data to allow for further adaptations and refinement [24].

### Setting and context

This study was conducted at the Faculty of Health, Medicine and Life Sciences (FHML), Maastricht University, the Netherlands. All FHML programmes have student-centred, integrated curricula that use a problem-based learning approach. We focused on three undergraduate programmes: Medicine, Biomedical Sciences and Health Sciences. All curricula are organised into thematic courses of 4–10 weeks. Unique teacher teams are responsible for the design, implementation and evaluation of the different courses. Chaired by a course coordinator, these teams consist of 4–7 members from a variety of academic disciplines, including clinical, social and basic sciences (e.g. physiology and anatomy).

### Participants

We recruited participants using purposive, maximum variation sampling [25] to obtain a multitude of perspectives from a diverse range of teachers [26]. We sampled 17 interviewees (9 male, 8 female; 7 biomedical scientists, 6 social scientists, 4 clinicians) who were part of teams that were responsible for various first- and second-year courses from different programmes: five from Biomedical Sciences, six from Health Sciences and six from Medicine. Following team research protocols from the educational [27] and healthcare [28–30] realms, we sampled one member per team who had at least one year of experience in this team. Participants varied with respect to gender, age (mean age: 47.7, age range: 31–62, SD = 8.2), professional background and formal team role. All participants were University Teacher Qualification certified [31]. From earlier research we knew that the selected teachers were members of teams that worked in an integrated (11), framework-guided (4), and fragmented (2) manner, yielding higher and lower levels of team learning [21]. Consequently, a breadth of team types were represented in our sample, thereby offering richness of information. Driven by a rather broad study aim and cross-case analyses among individuals, we achieved information power [32] after 17 interviews. Although a thorough analysis of 15 interviews offered sufficient insight into the factors that enable and inhibit team learning behaviour’, we conducted two more interviews with two additional clinicians to test our themes for controversial thoughts: no new information was obtained.

### Data collection

Interviews were conducted between November 2017 and March 2018 using an interview guide and accompanying vignettes (see also Meeuwissen et al. [21]). Both were developed using sensitising concepts from the team learning literature [13, 17, 33]. The interview guide included questions about team-task fulfilment, social cohesion, personal values in teamwork, and the role of the organisation in team learning. The vignettes mirrored Van den Bossche et al.’s [17] definitions of team learning behaviour’: *sharing *knowledge and experiences*, co-constructing* knowledge by engaging in shared information, and *constructive conflicts *that include negotiations leading to shared agreements based on integrated views. Moreover, they prompted participants to reflect on current and preferred team learning behaviour and on what they perceived as factors enabling and inhibiting it. After a test interview, we refined some questions and we continued making minor revisions (e.g. question order) throughout the data collection process, optimising the guide. Interviews lasted approximately 60 min, were audio-recorded, transcribed verbatim and anonymised.

### Data analysis

We performed a stepwise type of thematic analysis known as ‘template analysis’ [34]. First, we formulated broad, a priori codes, drawn from previous team learning research (e.g. social cohesion) [19] to organise our initial template (see Appendix I in the Electronic Supplementary Material) [34]. Consequently, the first author and a student-assistant independently coded the interview transcripts and discussed their codification to resolve discrepancies. Besides deductive, a priori coding, they used inductive coding to identify additional influences such as the role of educational philosophy. They merged and renamed codes, deleted redundant codes and identified code linkages. The focused template developed after a succession of hierarchically structured templates was iteratively applied to the data. A team learning researcher supported our final interpretation after having read three prototype interviews alongside the template. After this process, researchers discussed and negotiated code linkages to ultimately reach agreement on the three overarching themes and their subthemes. We managed our data using ATLAS.ti 8 (Scientific Software Development GmbH, Berlin, Germany).

### Reflexivity

The research team included a PhD candidate with a medical background (SM), two educational scientists—one working in Business and Economics (WG) and one in Health, Medicine and Life Sciences (IW)—and the FHML Institute for Education scientific director with a background in physiology and education (MoE). SM, who was trained and experienced in interviewing, conducted all interviews. Using their diverse backgrounds, the team discussed the main results and reached agreement on themes and subthemes. Due to managerial relationships, IW and MoE were not allowed access to transcripts. They recognised the main results from their own teacher-team experiences.

### Ethical considerations

Ethical approval was obtained from the Dutch Association for Medical Education Ethical Review Board (NVMO ERB-948). Participation was voluntary and all participants gave informed consent.

## Results

We identified three overarching themes reflecting the factors that enabled and inhibited team learning by teams with teachers from different disciplines: i) alignment/misalignment with the educational philosophy, ii) leadership practices and iii) involvement in organisational processes. Each theme comprised subthemes (factors) that were related to the: i) individual team members (personal attributes, tendencies and motivation); ii) team (vision, responsibility and reflection); and iii) organisation (decision-making, support and learning opportunities). Tab. 1 presents an overview. In the following, we will describe the themes identified, illustrated by representative quotations^1^ from interviewees.

**Table 1:** Overview of the main themes and subthemes-related to the individual, team and organisation-representing the factors that enable and inhibit team learning by teams with teachers from different disciplines

Themes and subthemes	Enabling	Inhibiting
*Alignment/misalignment with educational philosophy*		
Individual: attributes	Respect and appreciation for other disciplines, curiosity, being change-oriented, student-centred education mindset	Little appreciation for other disciplines, little experience in education, preaching to the converted, showing self-assertion, no student-centred mindset
Individual: tendencies	Ability to listen, to think outside the limits of disciplines and to work together in a creative and constructive way	Being stuck in routines, being inflexible
Individual: motivation	Motivated mainly to be involved in education and challenge oneself. Course content should be in line with members’ own background	Motivated merely to comply with boss’s demands, create visibility for own department, and have educational roles on CV
*Leadership practices*		
Team: vision	Shared team vision, sense of team identity, shared responsibility and commitment	Lack of shared course vision, lack of clear team task
Team: responsibility	Shared team leadership with shared responsibility and decision-making	Leader feeling individually responsible and making decisions alone
Team: reflection	Sharing of reflections on team functioning and acting upon reflections	No discussions on team functioning
*Involvement in organisational processes*		
Organisation: decision-making	Offering possibilities for involvement in change and choices in curriculum development. Having a say in the selection of team members	No sharing of or feedback on political or financial choices regarding education. No say in the composition of the team
Organisation: support	Help from peers, educational support staff and departments in educational tasks and functioning in education through interaction	No experience and knowledge to reach out for suitable educational support, dialogues and discussions
Organisation: learning	Opportunities to learn from others how to ‘do and think’ education in the organisation to create both team learning opportunities and a sustainable workforce	No sense of a sustainable workforce for teacher teams. No opportunities to learn from others

### Alignment/misalignment with the educational philosophy

Teachers of the various HPE programmes mentioned the importance of individual alignment with the educational philosophy prevailing at their institute (i.e. student-centred, integrated education using a problem-based learning approach). Moreover, they stressed that team learning for integrated education required individuals to be flexible, change-oriented, inquisitive and respectful of other disciplines. Such personal attributes created opportunities to share knowledge in interdisciplinary discussions, ultimately leading to shared agreements. If teachers embraced an integrated learning and teaching philosophy, their perspectives more easily aligned, which facilitated team learning:

*I [basic scientist] can make a beautiful clinical case from a textbook, and then a clinician will say ‘that may be the case in theory, but I never see that in practice.’ So, I do not want to present that case to our students. Also, the other way around, a clinician can have respect and appreciation for the fact that basic scientists can add knowledge that you do not use on a daily basis in the clinic but what we believe is important for students to take along* (Interview 17).

According to teachers, however, some individual tendencies did not fit well with this philosophy and hindered team learning. Behaviours perceived to work counterproductively were: ‘preaching to the converted’, showing self-assertion, and having low self-efficacy and little experience in education. Similarly, tendencies to be stuck in personal, often disciplinary routines and unable to see one’s task as a team effort due to a traditional soloist teacher perspective were regarded as unhelpful. Enabling tendencies, by contrast, were the ability to listen, think beyond disciplines and work together constructively.

With respect to teachers’ motivation to join a specific interdisciplinary course team, course content was considered key. More specifically, to enable fruitful discussions and negotiations, course contents had to match teachers’ expertise. Other motivational factors perceived to enable team learning were: intrinsic interest in education (e.g. ‘I have always been keen on working in education’) and personal development goals (e.g. ‘I like to challenge myself’). Motivational threats we identified were advocacy (e.g. ‘having an interest in departmental visibility’), career positioning (e.g. ‘career promotion requires educational roles on CV’) and compliance (e.g. ‘when someone is asked to be in this team by his/her boss cause no one else seemed appropriate’).

### *Leadership practices*

Leadership practices were mentioned to be important to the team’s vision, responsibility and reflection. One participant explained that when a shared vision was absent, no clear task allocation took place, which negatively affected team processes. Most participants agreed that shared decision-making led to feelings of shared responsibility and commitment to both the course as a whole and the corresponding interdisciplinary team process. As a result, team tasks were approached from a ‘we’ instead of an ‘I’ perspective. Such a shared team leadership approach was often considered organic:

*I believe that in this academic environment leadership is, well, we are all independent professionals [...] I rarely see a boss saying, this is how it needs to be done. It’s a lot of [pauses] doing things together (Interview 10).*

In contrast, some participants described how team learning was obstructed when dominant leaders were at the helm. Typically, these leaders felt they were responsible for everything, often failing to address team and individual functioning. The following quote illustrates how a team leader preferred to avoid conflict and remain silent when confronted with poor performance:

*I try to be nice in the beginning [when a team member demonstrates poor performance]. Because I think, well, if we end up with a fight in this teacher team, that would be really unpleasant. But eventually I think ‘up to here, and no further.’ And then I try to let such a person withdraw himself. I will hint that it really needs to stop at a certain point (Interview 3).*

Finally, many teachers pointed out that team reflection was an area needing improvement to enhance team learning. A lack of such reflection contrasted sharply with the learning practices taught to students: *‘We ask a lot of feedback and reflection from our students, but we are not really good at it ourselves.’* *(Interview 10).*

### Involvement in organisational processes

Various teachers stressed the importance of being involved in organisational decision-making. Some felt decisions, for instance to merge existing courses or construct a new integrated course, were imposed from above. For others, financial or political management choices created personal uncertainties, dissatisfaction and even frustration about having to work with colleagues from other disciplines. The following teacher did not feel in a position to voice concerns, causing emotions to prevail which hindered team learning:

*‘It is like a forced marriage, in which I respect and appreciate my partner, my spouse, and sit through the wedding, so to speak, because there is no point in arguing. It won’t do students any good’ (Interview 13).*

Next, a vast majority of teachers mentioned that, for team learning to happen, they needed to be able to co-select fellow team members based on their interest in education. Current application procedures were to select team members based on relevant expertise or previous educational performance or experience. To provide valuable input, however, teachers felt they needed to be able to ‘think education’ rather than ‘think their discipline’ and educational institutes had a responsibility to help them develop that skill. Teachers also emphasised the importance of dialogues and educational goal setting together with the wider community of peer teachers, educational support staff and department colleagues:

*I think that many [team leaders and members] barely know how they perform, why they do it that way, why they are doing it right, and why, perhaps, they are not.—It would be nice if such things are just freely discussed, really (Interview 2).*

To ensure a sustainable workforce and team success, some interviewees suggested that the organisation should appoint junior teachers, possibly aided by a mentor, so that they can learn how to work in an interdisciplinary way and create integrated education:

*This whole climate of education—it takes years to understand, to be in that structure. The young blood [new teachers] we have in our team, they know nothing, they just sit and stare.[…] they can only say something from their discipline (Interview 16).*

## Discussion

Simply putting different disciplines together for integrated HPE has shown to not automatically result in integrated teams, working interdisciplinarily [21]. The present study has offered insight into individual team members’ perceptions of factors that enable or inhibit learning by teams with teachers from different disciplines. Factors were related to the individual, the team and the organisation: personal alignment with the prevailing educational philosophy, shared team leadership practices and involvement in the organisation were perceived as important enablers by teachers working with different team approaches (see Meeuwissen et al. [21]). We therefore conclude that teachers, regardless of team functioning, have similar perceptions of interdisciplinary team learning. These findings could offer a first step in facilitating interdisciplinary teacher teams in HPE.

It is generally accepted that gathering individual experts does not necessarily guarantee successful teamwork [35]. Our results uncovered meaningful attributes and tendencies of individual members in integrated HPE. Ideally, clinical, basic and social sciences teachers should be motivated to working in integrated education and share similar values, despite their distinct professional backgrounds. This need also became evident in a study involving clinical postgraduate teachers, where impediments arose because teachers valued shared clinical performance goals to the neglect of shared educational visions [36]. A teacher team without a clear, shared vision is merely a group of individuals and cannot be considered truly interdisciplinary. High-level team learning requires members to acknowledge different views and engage in negotiations [15, 18]. If teachers from distinct disciplines do not share the educational philosophy, reaching final agreements on integrated education will be difficult.

Another enabling factor was a shared leadership capacity. Although traditional, hierarchical views on leadership are still pervading today’s healthcare professions [37], Day et al., too, concluded that a single leader cannot possibly envision all solutions, especially not in highly complex tasks, such as the development of integrated education [38]. Moreover, we found that dominant leaders, who felt strong personal responsibility for everything, were perceived to hinder team learning. Our study therefore moves beyond Day et al.’s theoretical assertions [38] by demonstrating that, in participants’ view, a traditional leadership style frustrated team dynamics, whereas a shared leadership approach promoted team learning.

Room for involvement in educational decision-making and teacher team composition, support through interaction across the organisation, and opportunities to learn about teamwork were deemed important. Sometimes, when teachers were unsatisfied with their team composition, rather than sharing this frustration or raising questions or concerns, they chose to remain silent. A lack of psychological safety could explain this behaviour [39], as silence could protect the individual [40]. However, this lack can simultaneously harm team performance since disengagement in discussions may cause suboptimal social interaction [40]. Our research findings not only point to a possible culture of silence in HPE, but also that this could fuel individual psychological distress because of suppressed emotions.

Lastly, interviewees suggested that team reflections needed improvement. Team reflexivity, a team’s reflection and subsequent action, is said to promote team learning and adaptation to reach the team’s goals [20, 41]. Notwithstanding this, team reflexivity appeared to be a bridge too far in our study, a finding that resembles those of other studies in education [42] and clinical teaching teams [36]. We see merit in exploring opportunities and interventions to increase psychological safety and team reflection in HPE. Edmondson has argued that a culture in which people are encouraged to ask questions, learn from mistakes, and try out creative and novel things together can feed team learning processes [39]. Similarly, teacher teams in HPE could benefit from such a learning culture in which people are not only flexible, adaptive and willing to discuss, but also work in an academic environment where they can and should continuously learn and share knowledge [43, 44].

The three main themes identified in the current study underscore the importance of having a sense of ownership, and being committed to the educational organisation HPE teachers belong to. Previous research has suggested that these factors contribute to continuous quality improvement in medical education [45]*.* Hence, just as the separate disciplines in the ‘Shortness of Breath’ course need to become more integrated, likewise teachers from various disciplines should feel integrated into their team and the educational institute they belong to.

### Methodological reflections and future research

We consider it a strength that we were able to identify themes that individual team members shared across a wide range of disciplines, HPE programmes and team approaches. However, future research should also explore individual perceptions *within* teams to discover whether all team members, too, share similar perceptions. Our variegated sample allowed for a comprehensive understanding of the organisational culture as well as general enablers and inhibitors of interdisciplinary team learning. Since our study was undertaken in one institute, findings may not be transferable to other settings due to structural and cultural differences. We therefore invite future researchers to compare teams from several institutes. Next, this study examined what teachers perceive as enablers/inhibitors, but we cannot know whether working on these factors will actually improve interdisciplinary team functioning in HPE practice. It would therefore be worthwhile to investigate how teacher team learning develops over time, and how to effectively promote this.

### Implications for practice

As a start, the teachers in the Introduction organising the ‘Shortness of Breath’ course could benefit from the explication of the integrated, student-centred education philosophy so that they can align their course vision accordingly. In general, when organising integrated curricula, HPE institutes must realise that interdisciplinary teams require more from individual members than monodisciplinary teams do. HPE institutes could pay attention to the themes identified, for instance by investing in staff who take a genuine interest in education and their school’s prevailing educational philosophy. This specifically holds for the HPE setting since teachers often participate in education, research and the organisation of their daily professional work [7]. In addition, HPE institutes could offer faculty development courses aiming to increase awareness of shared leadership, thereby moving away from the prevailing traditional thoughts on healthcare leadership [37]. Implementing courses with reflective approaches could help to acquire the right mindset [46]. Such courses could also empower HPE leaders to define a clear team vision, adapt to shared decision-making and responsibility, and encourage team reflection. They will be most effective when offered just in time, that is, *during* leadership activities [47]. Lastly, an open culture and peer dialogues on HPE seem important to be able to learn from one another besides ongoing dialogues on expertise information in healthcare. By introducing interdisciplinary mentoring systems for teaching staff, organisations can offer teams educational guidance and support in exchanging didactical information, and create room for involvement in educational management.

## Conclusion

If HPE wishes to transition from a traditional, monodisciplinary education approach towards interdisciplinary teams that develop integrated education, teachers’ alignment with the institute’s educational philosophy, shared team leadership and involvement in the organisation are key. This study suggests that the traditional barriers between disciplines, however high, are not insurmountable when paying explicit attention to how specific personal, interpersonal and organisational factors contribute to interdisciplinary teacher teams.

## Supplementary Information

Appendix 1. Initial template, derived from previous research findings on team learning

## Footnotes

1 For purposes of enhanced coherence and legibility, quotes were subject to light editing before journal submission. The authors, however, based their analysis on the original, untidied-up transcriptions. If necessary, the original documents can be requested from the first author.
