# Supplementary material for: Working beyond disciplines in teacher teams: teachers’ revelations on enablers and inhibitors
Source: Perspect Med Educ. 2020 Dec 22;10(1):33–40. doi: 10.1007/s40037-020-00644-7 (PMC7809069; doi:10.1007/s40037-020-00644-7)
Supplement: Supplementary file 1 — Appendix 1. Initial template, derived from previous research findings on team learning [file 40037_2020_644_MOESM1_ESM.docx]

**Appendix 1. Initial template, derived from previous research findings on team learning**

*Motivation*

*Personal (team) values*

*Personality traits*

*Interpersonal beliefs*- Group potency
- Social cohesion
- Task cohesion
- Psychological safety

*Systems thinking*- Social interdependence
- Task interdependence

*Identity*- Professional identity
- Team identity

*Team reflexivity*
